# Crystal-Phase Engineering of Nanowires and Platelets of K_*x*_IrO_2_ for Efficient Water Oxidation

**DOI:** 10.1021/acsmaterialsau.5c00127

**Published:** 2025-10-15

**Authors:** Rachael Quintin-Baxendale, Maria Sokolikova, Yemin Tao, Evan Fisher, Nagaraju Goli, Haoyu Bai, James Murawski, Guangmeimei Yang, Veronica Celorrio, Caiwu Liang, Reshma R. Rao, Ifan E. L. Stephens, Cecilia Mattevi

**Affiliations:** † Department of Materials, 4615 Imperial College London, London SW7 2AZ, U.K.; ‡Diamond Light Source Ltd ., Harwell Science and Innovation Campus, Chilton, Didcot OX11 0DE, U.K.; § Department of Chemistry, University College London, 20 Gordon Street, London WC1H 0AJ, U.K.; ∥ UK Catalysis Hub, Research Complex at Harwell, Rutherford Appleton Laboratory, Harwell Oxon, Didcot OX11 0FA, U.K.; ⊥ Grantham Institute—Centre for Climate Change and the Environment, Imperial College London, South Kensington Campus, London SW7 2AZ, U.K.

**Keywords:** iridium oxide, thermal annealing, crystal phase engineering, oxygen evolution, water electrolysis

## Abstract

IrO_2_ is one of the most widely investigated electrocatalysts for oxygen evolution reaction in an acidic environment. Increasing the mass activity is an effective way of decreasing the loading of Ir, to ultimately reduce costs. Here, we demonstrate the crystal-phase engineering of two different potassium iridate polymorphs obtained by designing a selective solid-state synthesis of either one-dimensional K_0.25_IrO_2_ nanowires with a hollandite crystal structure or two-dimensional KIrO_2_ hexagonal platelets. Both structures present increased specific and mass electrocatalytic activities for the water oxidation reaction in acidic media compared to commercial rutile IrO_2_ of up to 40%, with the 1D nanowires outperforming the 2D platelets. XANES, extended X-ray absorption fine structure, and X-ray diffraction investigations prove the structural stability of these two different allotropes of KxIrO_2_ compounds upon electrocatalytic testing. These low-dimensional nanostructured 1D and 2D KxIrO_2_ compounds with superior mass activity to commercial IrO_2_ can pave the way toward the design of new electrocatalyst architectures with reduced Ir loading content for proton exchange membrane water electrolyzer (PEMWE) anodes.

## Graphical abstract

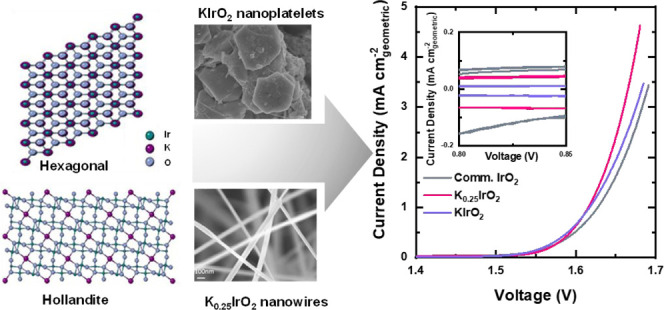

## Introduction

Using hydrogen as both a clean energy source and vector offers promise due to its high energy density and sustainable production possibilities, such as water electrolysis.^1,2^ Currently, according to International Energy Agency Global Hydrogen Review,^3^ water electrolysis is responsible for ∼0.5% of hydrogen production worldwide, with the majority of the globe’s hydrogen formed via steam reformation of fossil fuels.^4^ While proton exchange membrane water electrolysis (PEMWE) is an attractive technology for green hydrogen production, scaling it to the terawatt level is presently challenging due to the limitations of the system. The oxygen evolution reaction (OER) at the anode is a multistep reaction which requires a large thermodynamic overpotential, in turn reducing the overall efficiency.^5,6^ The scarcity of Ir means that only decreasing the loading of Ir by a factor of at least 10 would be required to scale up the PEM electrolysis technology.^7–9^

Proton exchange membrane electrolyzers (PEMWEs) offer higher pressure operations, voltage efficiencies, H_2_ purity, current density, and reduced ohmic losses, combined with a smaller stack design, compared to traditional alkaline electrolyzers.^10,11^ However, these utilize platinum group metal catalysts as they have the highest efficiency and are able to withstand the very harsh acidic and oxidizing conditions experienced at the anode.^12,13^ Currently, the commercially available catalysts for the OER are iridium-based, usually IrO_2_, with both high electrocatalytic activity and stability.^14^ The scarcity of Ir, combined with the current high loading levels required on the anode (2 mg cm^–2^), makes the PEMWE very expensive.^15^ Nanostructuring the catalysts and changing the morphology of IrO_2_ offer promise for increasing the surface area and the number of active sites per geometric footprint of the electrode, and this approach does not necessarily require the use of other materials which are not stable within the PEMWE environment and allows for the direct lowering of required loadings.^16–18^ Investigations have shown the difference in activity and stability based on the crystallinity of IrO_2_: while highly crystalline, rutile IrO_2_(110) offers good activity and high stability,^19^ and amorphous IrO_*x*_ gives better activity and lower stability.^20–22^ In our previous work, we show that this activity difference in amorphous IrO_*x*_ compared to rutile IrO_2_ is due to the higher site density of redox centers for the amorphous example, but the lack of crystallinity causes instability.^20^ It has been reported that the most active and stable amorphous IrO_*x*_ type has an hollandite-like structural domain with a monoclinic unit cell, and the ratio here between the corner- and edge-sharing IrO_6_ octahedra is different from the one of the rutile phase.^23,24^ Ideally, a catalyst, therefore, will have the benefits of the hollandite-like structural domain with a monoclinic unit cell, typical of the semiamorphous IrO_*x*_ with an elevated stability induced by an extended crystalline structure.

Currently, high-surface-area nanoparticles are used as catalysts for the OER, but they are prone to dissolution, causing redeposition to form larger particles.^25^ The design of one- and two-dimensional (1D and 2D) crystalline nanostructures in the form of nanowires, nanorods, nanotubes, or platelets could offer high surface areas and thus a high number of active sites, which can lead to high specific activity, preserving stability during cycling.^26^ In specific, 2D van der Waals structure nanoplatelets^27–36^ can offer structural stability and high surface area to be used as electrocatalysts. However, an ongoing challenge is the ability to synthesize 2D platelets of IrO_2_ as it is a non-van der Waals material. Previous works have demonstrated the synthesis of IrO_2_ nanoplatelets, either hydroxylated or stabilized, using Na ions. The electrocatalytic activity for O_2_ evolution remained unknown, with sodiated samples obtained via a solid-state synthesis at 600 °C for 5 days and hydroxylated samples obtained through acid immersion.^37–39^ Additionally, hollandite K_0.25_IrO_2_ nanoribbons^40^ and nanorods^41,42^ have previously been synthesized at temperatures above 750 °C to obtain high crystallinity. While these materials demonstrated promising electrocatalytic activity, they often suffered from heterogeneous stacking, partial phase impurities, and restricted accessibility of active sites.

In this work, we demonstrate the tunable formation of KIrO_2_ platelets as well as hollandite K_0.25_IrO_2_ nanowires with improved electrocatalytic activity compared to an optimized commercial IrO_2_. We have designed a precisely controllable solid-state synthesis process with either KIrO_2_ platelets or K_0.25_IrO_2_ nanowires with phase-pure hollandite tunnels. The synthesis of 2D hexagonal-layered KIrO_2_ platelets, with thickness in the nanometer range, is reported for the first time, while the production of 1D hollandite K_0.25_IrO_2_ nanowires shows enhanced electron/ion transport, exposes more catalytic interfaces, and improves structural stability, leading to superior electrochemical performance compared to earlier hollandite K_0_._25_IrO_2_ materials.^42,43^ The electrocatalytic activity and stability of hollandite structures were tested using a rotating disk electrode (RDE) within a 0.1 M HClO_4_ electrolyte and compared with optimized commercial IrO_2_; the results show improved activity, with lower overpotentials of 0.41 and 0.42 V at 1 mA cm_geometric_^–2^ for K_0.25_IrO_2_ and KIrO_2_, respectively, compared to 0.43 V for Commercial IrO_2_. This study paves the way toward the optimization of electrocatalytic activity of KxIrO_2_ compounds for use in PEMWEs, showing the advantage of using nanostructured, highly crystalline materials versus commercial IrO_2_.

## Results and Discussion

An efficient solid-state synthesis approach was developed to controllably produce 1D and 2D potassium iridate structures. The schematic in Figure 1a,b shows that in a typical preparation of intermediate compound, a layered K_*x*_Ir_*y*_O_*z*_ was formed via 2 heating cycles (Figure S1), the last of which determining the crystallinity and dimensionality of the structure.^44^ K_2_CO_3_ and IrO_2_ were used as precursors and ground and pelletized (Figure 1a). They were then annealed under Ar conditions within the temperature range of 750–780 °C two times, with an intermediate step of grinding and pelletization (Figure 1b). Further details on the reduction in heat cycles required within this work, compared to the literature, can be found within the Experimental Section. The obtained compound has a composition of K_*x*_Ir_*y*_O_*z*_, and it is layered (Figure 1c,d) as revealed by X-ray diffraction (XRD) and scanning electron microscopy (SEM), respectively (Figures 1c,d). XRD investigation shows reflections at 12° and 24° which are characteristic of layered K_*x*_Ir_*y*_O_*z*_.^44^ Further peaks that are seen can be assigned to the SiO_2_ substrate (*), IrO_2,_ ^45^ and Ir metal,^46^ with the latter formed as byproducts of the synthesis. The morphology of the K_*x*_Ir_*y*_O_*z*_ compound (Figure 1c) consists of thin, regular sheets arranged in a layered structure, with the lateral size in the tens of microns range. This compound was very unstable in air and water, and it was beam-sensitive, likely due to the large amount of potassium in the structure (photograph in the inset of Figure 1c). Finally, the obtained K_*x*_Ir_*y*_O_*z*_ pellet underwent a final annealing step under controlled growth temperatures. At a temperature of 550 °C, the product attained the form of KIrO_2_ platelets (Figure 1e), while at the annealing temperature of 750 °C, the product was in the form of K_0.25_IrO_2_ nanowires (Figure 1f). The morphology of KIrO_2_ under different temperatures is shown in Figure S2.

**Figure 1:**
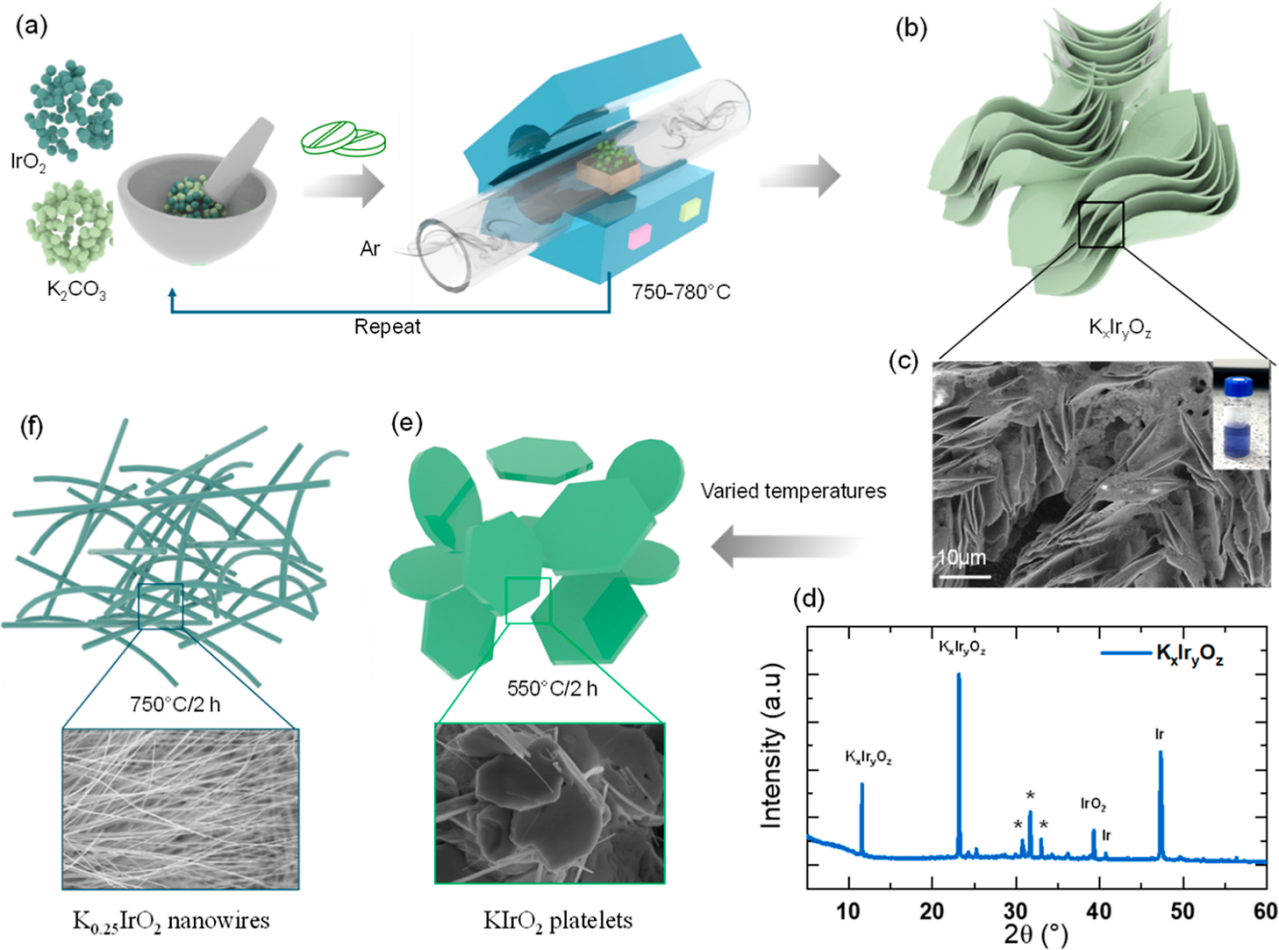
(a) - (e) Schematic depicting the synthesis procedure, including intermediate and final products. (d) XRD pattern and (c) SEM image of the KxIr_*y*_O_*z*_ intermediate compound. The inset of (c) shows the photograph of KxIr_*y*_O_*z*_ in water.

Figure 2 shows the morphology and structural properties of KIrO_2_ platelets synthesized at the annealing temperature of 550 °C. As shown in Figure 2a, the prepared sample showed well-defined nanoplatelets of approximately 800–900 nm in length, with the lateral thickness ranging from 50 —to 100 nm (Figure 2a). From EDS mapping (Figure 2c), we can infer that these platelets are composed of K, Ir, and O without any impurities. This stoichiometry suggests that iridium is in its 3+ oxidation state, which is confirmed from the X-ray photoelectron spectroscopy (XPS) fittings shown in Figures 2g–i and S4d. The Ir^III^ 4f_7/2_ peak is located at 61.47 eV, approximately 0.8 eV below the values observed for pure IrO_*x*_ samples.^47^ The O 1s spectrum (Figure 2h) similarly exhibits Ir–O lattice oxygen as the main component, with minor oxygen vacancy contributions, while the K 2p spectrum (Figure 2i) further verifies potassium incorporation, showing the expected K 2p doublet alongside a small peak attributed to the surface carbonate contribution. This can be explained by the small electronegativity of potassium present in the structure, serving to increase the electron density at iridium centers and causing a shift to lower binding energies.^43^ Additionally, transition electron microscopy (TEM) was required for the investigation of the microstructure due to the beam-sensitive nature of the sample, leading to the degradation of the platelets into very small nanoparticles. SAED diffraction patterns (Figure 2b) show a uniform hexagonal array of diffraction spots which have interplanar distances of 2.71 Å and 1.57 Å, matching the (100) and (110) planes, leading to relative spot intensities, in agreement with the simulated pattern of a tetragonal unit cell of KIrO_2_. Additional indications of the presence of KIrO_2_ include the closeness of the (100) and (110) intensities and the lack of half-period spots, which further suggest the unit cell to be hexagonal. HR-TEM images (Figure S3) display the hexagonal lattice of the KIrO_2_ platelets with a uniform *d*-spacing of 2.71A, agreeing well with the SAED data. EDS corroborates the stoichiometry of KIrO_2_ from the K, Ir, and O Kα and M peaks. The XRD reflections at 12.9° and 25.7°, respectively, match the KIrO_2_ *P*6_3_/*mmc* simulated reference pattern, thus having a hexagonal unit cell (Figure 2d). The lattice parameters can be estimated via indexing of the XRD pattern, giving *a* = 3.15 Å and *c* = 13.72 Å. Figure 2e,f shows that KIrO_2_ is made up of layers of edge-sharing iridium oxide octahedra, with the potassium ions positioned centrally within the interlayer spacing. This is the first time a hexagonal structure has been found in the KIrO_2_ stoichiometry.^48–50^

**Figure 2:**
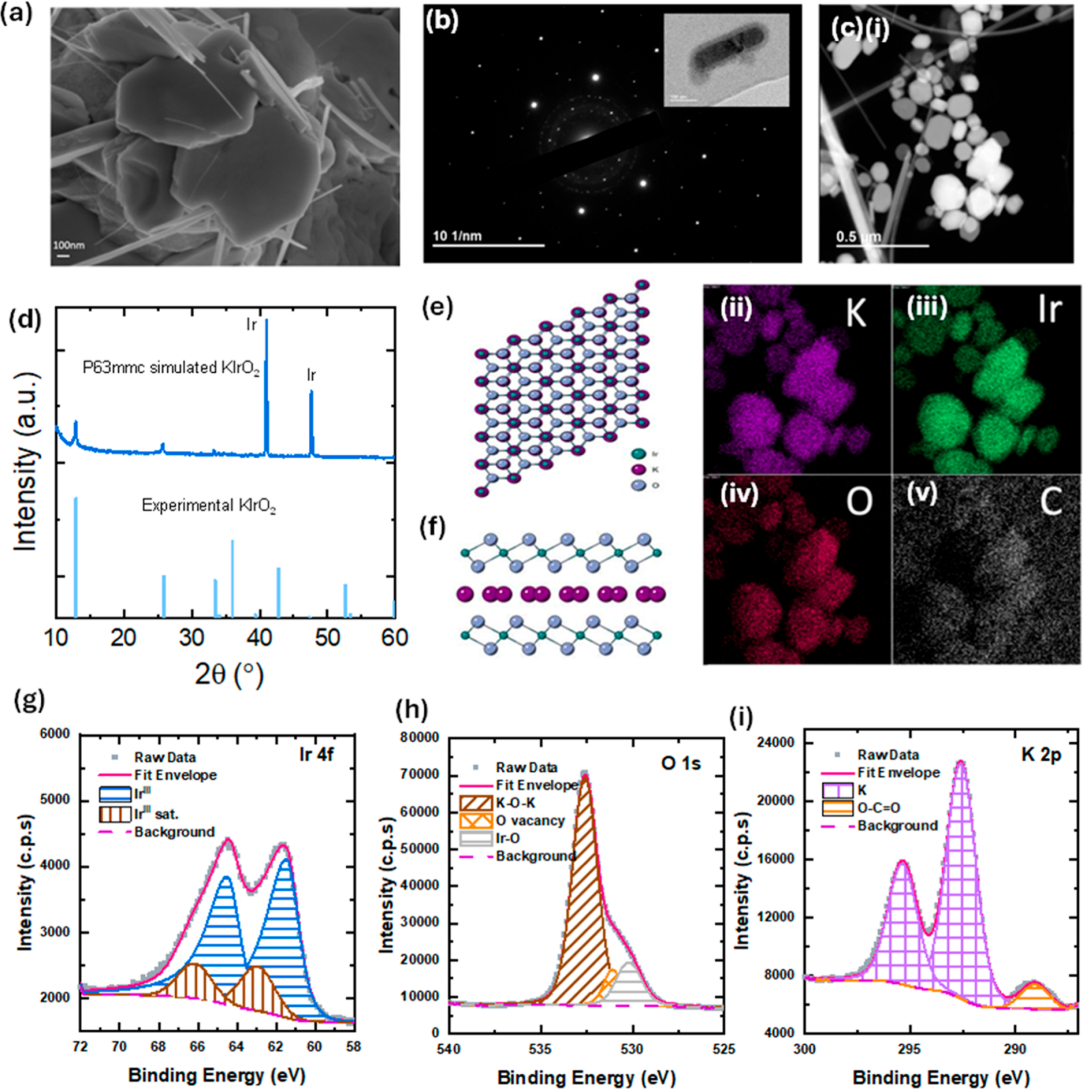
All portraying data for KIrO_2_ nanoplatelets after synthesis. (a) SEM image; (b) SAED data with inset; (c (i–v)) EDX mapping from TEM images, showing the presence of K, Ir, O, and C; (d) XRD pattern; (e,f) hexagonal crystal structure schematics (top view and side view). (g–i) High-resolution XPS spectra of Ir 4f, O 1s, and K 2p/Ir 4d of KIrO_2_ platelets. In g, the peak fitting is performed using only Ir^III^, corroborating the stoichiometry from EDS.

In a second case, we have thermally treated the intermediate compound at a higher temperature of 750 °C. Highly crystalline nanowires, with a diameter of ∼20 nm (Figure 3a), grown along the *c*-axis of the hollandite structure were obtained. Structural and compositional characterizations indicate the presence of K and the overall composition of the nanowires of K_0.25_IrO_2_. The stoichiometric ratio was inferred by TEM EDX analysis (Figure 3b) showing a K/Ir ratio of 0.25:1. This is further corroborated by XPS data (Figure 3i–k), where the area ratio of Ir^III^ to Ir^IV^ peaks in both Ir 4f and Ir 4d is shown to be 1:3, giving an equivalent oxidation state of +3.75. As in the nanoplatelet sample, the peaks were shifted approximately 0.8 eV below that of their pure IrOx counterparts, which is attributed to the presence of potassium.^43^ The nanowires appear to have a hollandite crystal structure (lattice parameters of *a* = 10.02 Å, *b* = 3.15 Å, *c* = 10.05 Å) and space group *I*12/*m*1,^42^ on the basis of SAED and XRD analyses. SAED patterns (Figure 3c,f) provide interplanar distances between the (020) and (002) planes^42,43,51^ of 5.02 Å and 1.57 Å, respectively, which match well with both the simulated (seen in the inset) and literature of hollandite structures.^42,43,51^ The HR-TEM images in Figure 3d,e also confirm the highly crystalline lattice behavior. The XRD pattern of the nanowires (Figure 3f), with reflections seen at 12.4°, 17.5°, 28.0°, 34.7°, 37.8°, 47.1°, and 57.3°, has further confirmed the hollandite structure. SEM images, such as in the example shown in Figure 3a, show the nanowires in a well-separated arrangement as they have been imaged from a dry powder in contrast to the bundles observed under TEM, where the nanowires were drop-cast from IPA solution. Lower magnification SEM images show the uniformity and highly dispersed nature of the wires (Figure S2f). The hollandite K_0.25_IrO_2_ structure is shown in the schematic (Figure 3g,h), with the central K ions surrounded by a distorted IrO_6_ octahedra channel. The distortion of the IrO_6_ octahedra is known to enhance the electrocatalytic activity for the OER in the crystalline IrO_2_.^52,53^

**Figure 3:**
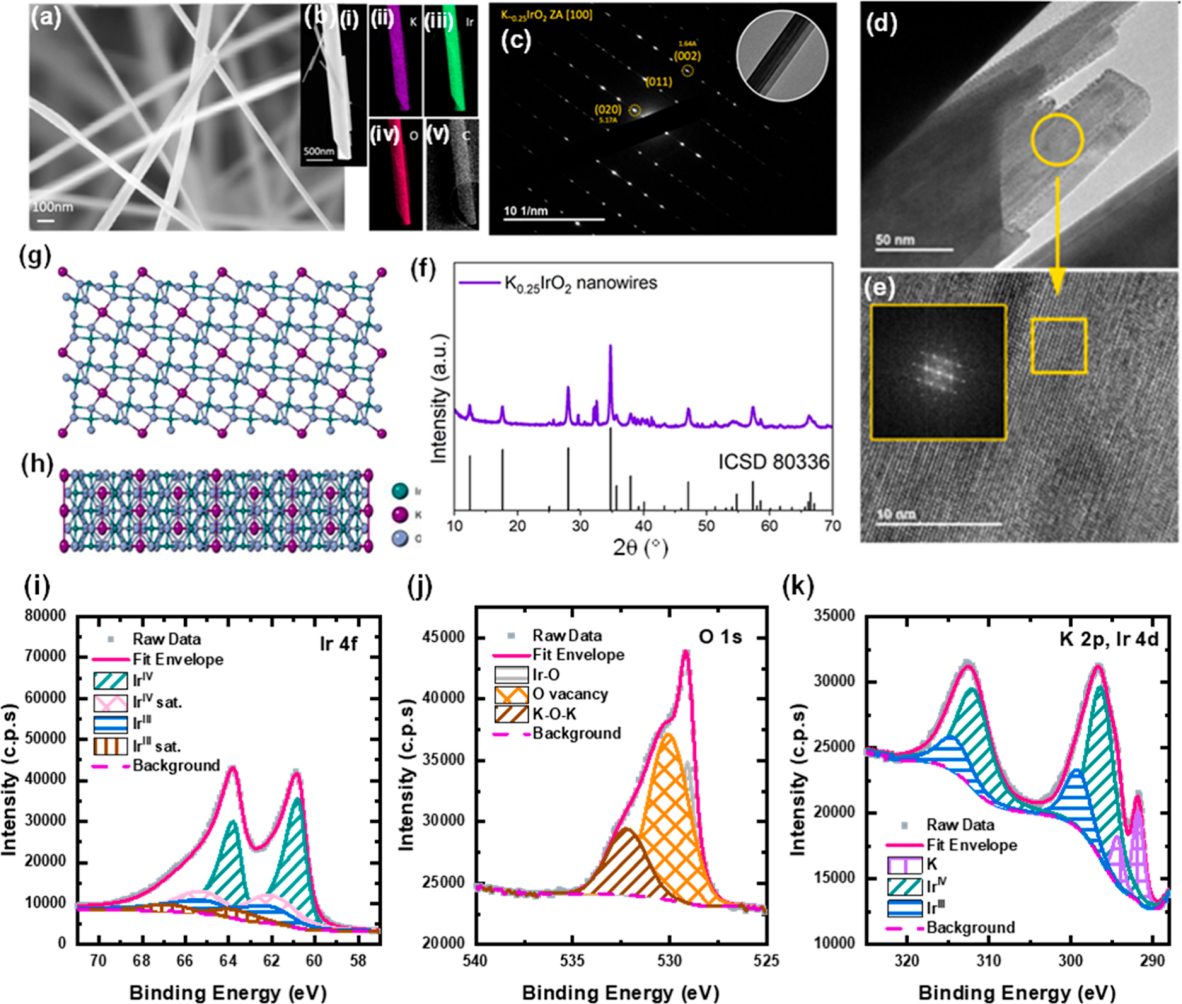
All portraying data for K_0.25_IrO_2_ nanowires. (a) SEM image; (b (i–v)) TEM image and EDX mapping; (c) SAED data; (d,e) HRTEM data with the SAED inset; (f) XRD pattern compared with reference card ICSD 80336;^43^ (g,h) crystal structure schematic (top view and side view). XPS fittings of (i–k) K_0.25_IrO_2_ nanowires. In (i,k), the area ratio of Ir^IV^ to Ir^III^ peaks is ∼3:1, corresponding to an overall iridium oxidation state of ∼3.75.

The electrochemical activity and stability were tested of both the nanowires and nanoplatelets and compared to a commercial IrO_2_ (5 nm nanoparticles, Alfa Aesar). A rotating disk electrode setup was used within an acidic electrolyte of 0.1 M HClO_4_ and a mass loading of 0.01 mg cm^–2^ (Figure 4). It is worth noting that the tested commercial IrO_2_ has been fully optimized for the OER, and further IrO_2_ catalysts were compared (Figure S4b) (Experimental Section). For all samples, double-layer capacitance was measured to determine the electrochemical surface area (Figure 4b, full figures shown in Figure S7). Figure 4a shows the linear sweep voltammetry (LSV) polarization curve of KIrO_2_, K_0.25_IrO_2_, and the commercial IrO_2_, cycled from 0.4 to 1.7 V vs RHE, after *iR* correction (Figure S9). To achieve a current density of 1 mA cm_geometric_^–2^, K_0.25_IrO_2_ nanowires achieve an overpotential of 0.41 V and KIrO_2_ platelets an overpotential of 0.42 V, outperforming the commercial IrO_2_ at 0.43 V. Additionally, at 1.65 V vs RHE, K_0.25_IrO_2_ achieves a current density of 2.38 mA cm_geometric_^–2^ and KIrO_2_ a current density of 1.92 mA cm_geometric_^–2^, again outperforming the commercial IrO_2_ at 1.61 mA cm_geometric_^–2^. The mass activities are compared in Figure 4d, with the mass activities of both outshining those of commercial IrO_2_. Specifically, the K_0.25_IrO_2_ catalyst has a superior mass activity of 27%. We can conclude that both KIrO_2_ and K_0.25_IrO_2_ have improved activity when compared to commercial IrO_2_. This is further shown by the iridium-specific activity, where the addition of potassium to the structure reduces the proportion of iridium and results in an increased performance (Figure S8). It is also clear that KIrO_2_ samples have exceptional area-specific (ECSA) activity but lack the number of active sites present in both commercial IrO_2_ and K_0.25_IrO_2_ nanowires, resulting in a slightly similar mass activity to the other samples. The stability of all three catalysts was then investigated (Figure 4c) by using the Accelerated Stress Test (AST) degradation method, based upon a benchmarking protocol previously reported by us,^55^ and analyzing the electrolyte post-test using ICP–MS to quantify the levels of Ir dissolution (Figure S11). Cyclic voltammetry (CV) scans between 1.2 and 1.7 V vs RHE at 600 mV s^–1^ for 15,000 cycles exhibited the extended cycling behavior (Figure S6). This was followed by a reconditioning step of 50 mV s^–1^ between 0.6 and 1.4 V vs RHE; then, the initial CV scans were repeated, and the percentage drop was reported (see Supporting Information for further details). These results show that the stability of commercial IrO_2_ appears higher, with an activity drop of ∼28%, compared to drops of ∼41% and ∼57% for K_0.25_IrO_2_ and KIrO_2_, respectively (Figure 4c). For ICP–MS, samples of the electrolyte were taken from the stability tests of K_0.25_IrO_2_, KIrO_2_, and commercial IrO_2_ at 3 regular intervals (Interval 01: after initial activity test; Interval 02: after 15,000 CV scans; Interval 03: after repeated activity test), and the data are shown in Figure S11. A sudden increase in concentration of Ir for commercial IrO_2_ was observed after the extended CV scans, and then a final concentration of 0.410 ppb of Ir after the final stability test was found. In comparison, after the initial activity tests, there is no major increase in Ir concentration for both KIrO_2_ and K_0.25_IrO_2_, and although the amounts of dissolved Ir are higher after the initial activity test, it appears that the dissolution of Ir has been suppressed compared to commercial IrO_2_ (Figure S11), with the final concentrations of 0.223 and 0.363 ppb for KIrO_2_ and K_0.25_IrO_2_, respectively. From these data, we can infer that the lower stability seen via higher activity losses of both KIrO_2_ and K_0.25_IrO_2_ is not due to high dissolution rates but likely due to the nanowires and platelets forming porous catalyst layers and therefore causing trapping of O_2_ bubbles within the structure and covering the active sites during the initial activity measurements or a change in the catalyst composition during cycling. To better understand the stability and increased OER in the KIrO_2_ and K_0.25_IrO_2_ nanostructures, we have investigated the material before and after electrocatalysis using XRD, extended X-ray absorption fine structure (EXAFS), and XANES (X-ray absorption near-edge structure) measurements.

**Figure 4:**
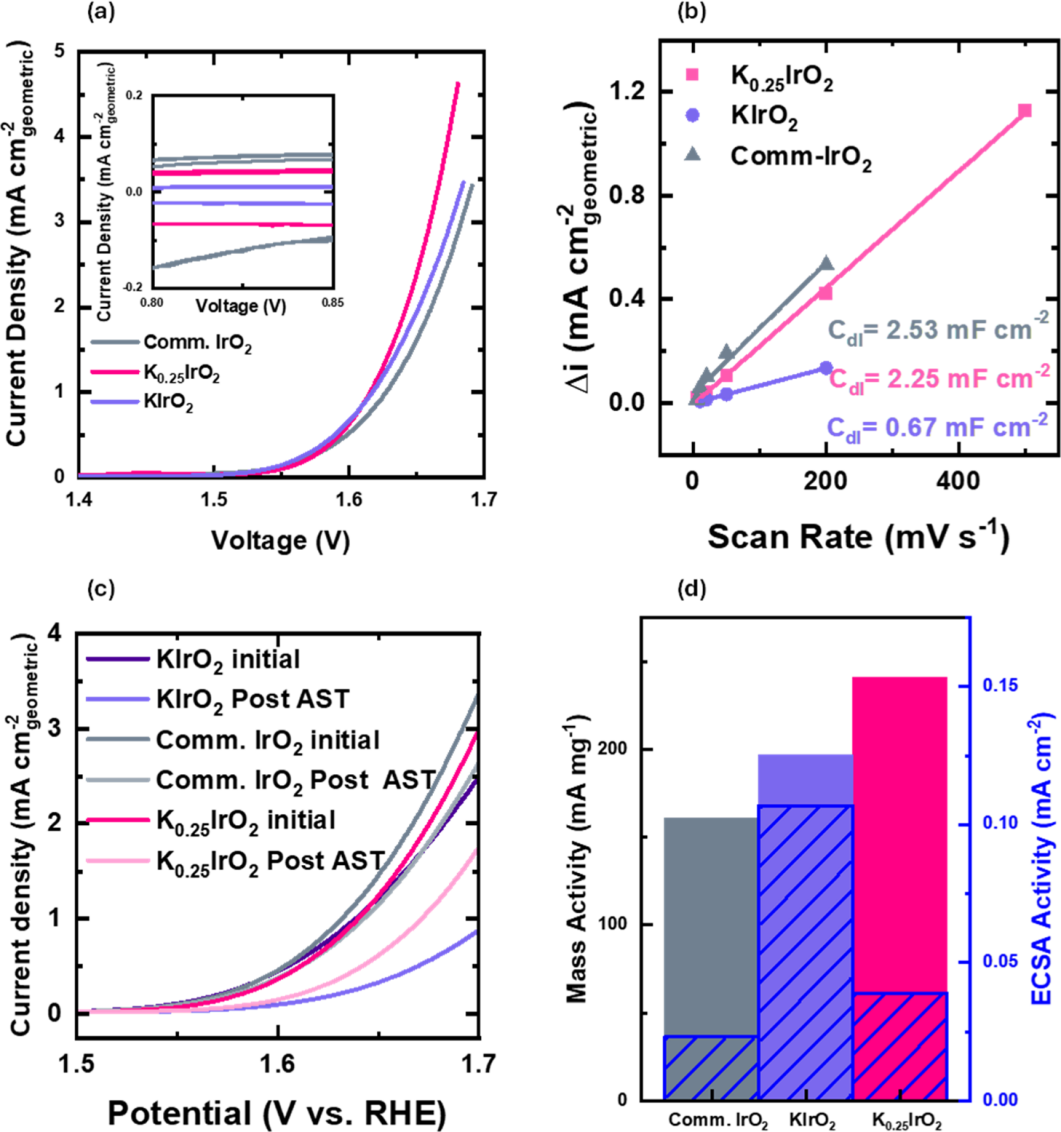
Electrochemical data collected for K_0. 25_IrO_2_, KIrO_2_, and commercial IrO_2_, all tested within 0.1 M HClO_4_, with mass loading of 0.01 mg cm^–2^ on the RDE. (a) LSV plot between 1.4 and 1.7 V vs RHE at 20 mV/s; inset: snapshot of the double-layer capacitance region for all 3 samples, taken at a scan rate of 50 mV s^–1^. Geometric activity is proportional to the mass activity of the sample; (b) double-layer capacitance measurements for K_0.25_IrO_2_, KIrO_2_, and commercial IrO_2_; (c) initial and post-AST activity for KIrO_2_, K_0.25_IrO_2_, and commercial IrO_2_; (d) mass activity calculated at 1.65 V vs RHE, with the corresponding activity normalized to the electrochemical surface area shown._._ All the data were corrected for the ohmic drop, and the total loading of iridium was 0.01 mg.^54^

Figure 5a shows the XANES spectra collected at the Ir-L_3_ edge of KIrO_2_ before and after AST. The white line position of KIrO_2_ shifts from around 11218.3 to around 11218.7 eV after AST, indicating an increase in the average Ir oxidation state from below 3+ to around 3+ (see Figure S12 for correlation between the white line positions and the oxidation state). The observation that the average Ir oxidation state of pristine KIrO_2_ is below the theoretical value predicted by stoichiometry can be attributed to the presence of some residual metallic iridium, as also supported by XRD results (Figure S10). During the AST test, this metallic iridium is expected to partly oxidize to iridium oxides, resulting in an increase in the average oxidation of iridium.^20^ The Fourier transform EXAFS spectrum of KIrO_2_ shows an interaction distance at around 1.6 Å, which corresponds to the Ir–O shell in KIrO_2_, while a higher intensity of signal at around 2–4 Å is typically related to different Ir–Ir shells (Figure 5b, see details of EXAFS data analysis in Table S1). The Ir–O shell signal is relatively lower than the Ir–Ir shell compared with typical iridium oxides,^20,56^ which might be attributed to the presence of the Ir metal phase, as also supported by XANES and XRD results. The change in the peak shape of EXAFS spectrum between 2 and 4 Å is probably due to the change resulted from the oxidation of metallic iridium into iridium oxides after AST. Given that the OER activity is higher for metallic iridium compared to IrO_2_ due to the electrochemical formation of an amorphous oxide layer on anodic polarization,^22,57,58^ we hypothesize that the decrease in catalytic activity following AST for KIrO_2_ is the result of oxidation of residual Ir metal in the structure. For K_0.25_IrO_2_, the XANES spectra (Figure 5c) show only a slight change in the white line peak positions (∼11219.1 and 11219.2 eV before and after AST, respectively), with the average oxidation state of ∼3.5 and 3.7 before and after cycling, close to the theoretical values of K_0.25_IrO_2_. Consistently, the EXAFS spectra before and after the AST test are similar, without obvious changes in the shape or peak positions, indicating a high structural stability of K_0.25_IrO_2_ during AST and, therefore, also chemical stability (Figure S10). Therefore, we can conclude that the decline in the apparent activity of KIrO_2_ and K_0.25_IrO_2_ after AST is not caused by chemical or structural changes in the materials but, as hypothesized earlier, indeed due to trapping of O_2_ bubbles^59^ caused by the porous morphology of the film. This would not be an issue within an electrolyzer system due to the continuous flow of water and gas through the PEMWE, where porous structures actually promote the passing of gases through the system.

**Figure 5:**
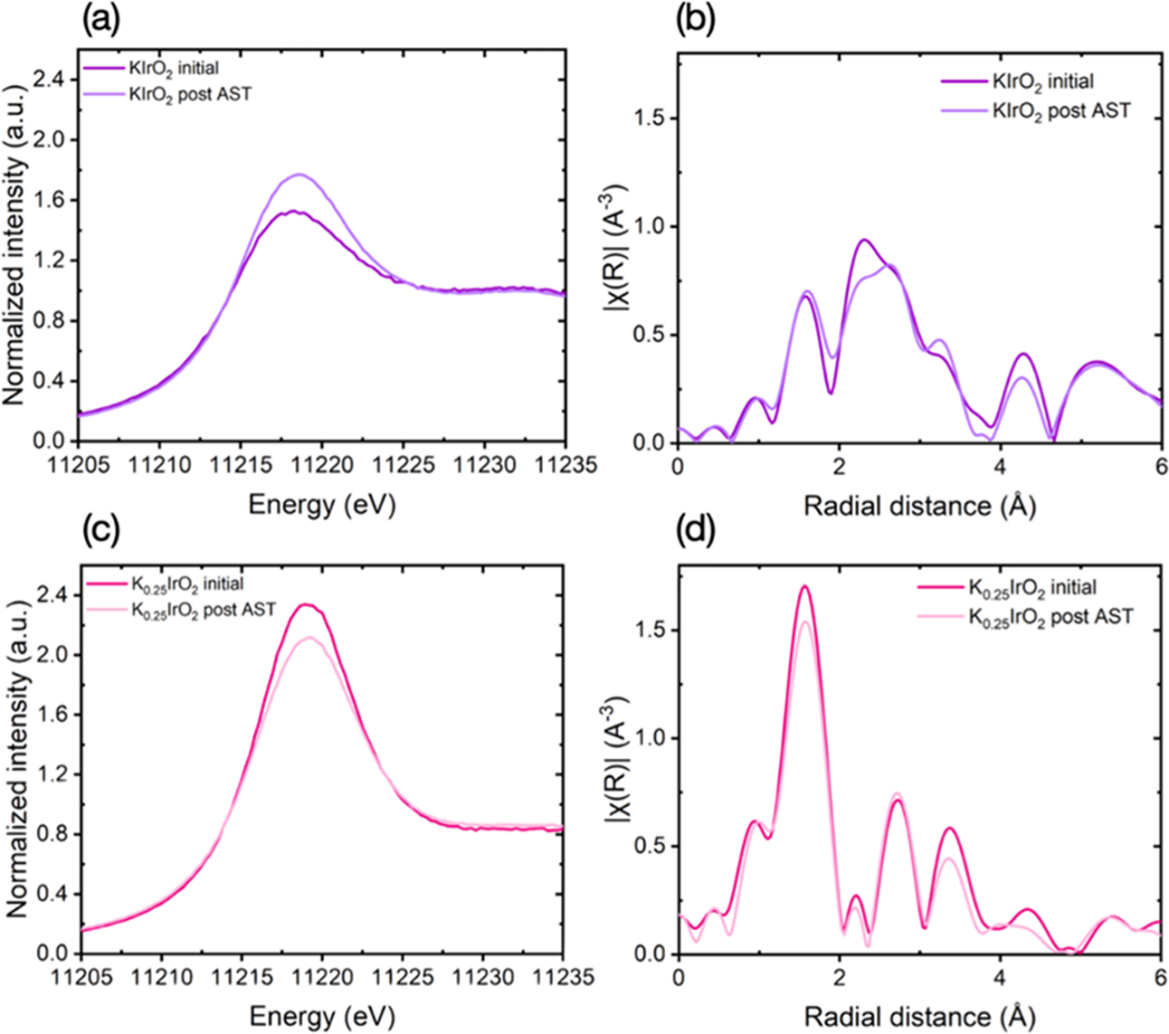
Ir L_3_-edge XANES region of (a) KIrO_2_ and (c) K_0.25_IrO_2_ as-received and post-electrochemical testing; *k* ^2^-weighted Fourier transforms of EXAFS spectra collected at the iridium L-edge of (b) KIrO_2_ and (d) K_0.25_IrO_2_ before and after AST.

## Conclusions

To conclude, a tunable, reproducible synthesis has been demonstrated that is able to selectively produce either novel KIrO_2_ platelets or highly uniform and crystalline K_0.25_IrO_2_ nanowires. Layered KIrO_2_ platelets present an hexagonal arrangement with sandwiched K ions, and K_0.25_IrO_2_ nanowires offer a highly crystalline hollandite structure, with channels of distorted IrO_6_ octahedra surrounding central K ions. Both structures present improved specific and mass electrocatalytic activities for oxygen evolution compared to commercial IrO_2_ with suppressed Ir dissolution. XANES and EXAFS investigations further prove the structural stability of these two different allotropes of the K_*x*_IrO_2_ compounds. Overall, the low dissolution rate and consequently high stability of both the K_*x*_IrO_2_ compounds are most likely induced by their high crystallinity and uniformity when compared to commercial IrO_2_, and therefore we have successfully achieved the benefits of both high activity and high stability. The solid-state synthesis of hollandite nanowires demonstrated here can lead to high crystallinity, material homogeneity, and crystal-phase tunability across gram scales at low temperatures. Both these low-dimensional nanostructured 1D and 2D K_*x*_IrO_2_ compounds with superior mass activity to commercial IrO_2_ can pave the way toward the design of new electrocatalyst architectures with reduced mass loading.

## Experimental Section

### Materials

Iridium­(IV) oxide (IrO_2_, 99%, 50 nm nanoparticles), iridium­(IV) oxide (IrO_2_, 99%, 5 nm nanoparticles), and potassium carbonate (K_2_CO_3_, 99%) were purchased from Alfa Aesar. Isopropanol (IPA), perchloric acid (HClO_4_, 70 wt %), and Nafion solution (5 wt %) were purchased from Merck, Germany. All chemicals were used as received. Water used in all experiments was deionized.

### Synthesis of K_*x*_Ir_*y*_O_*z*_

The synthesis of the layered potassium iridate intermediate compound is based on a solid-state reaction process. In a typical procedure, a 1:1 mixture of K_2_CO_3_ and IrO_2_ is ground by hand in an agate mortar for 30 min and then compressed in a 13 mm pelletizer under a pressure of 2 tonnes for 10 min. Then, the pellet is placed into an alumina crucible with a lid and calcined at 780 °C for 1 h under Ar flow. Once the pellet is cooled, the grinding and pelletizing process are repeated, and the pellet is calcined again at 750 °C for 1 h under Ar flow. The final product is left to cool and reground into a powder for characterization purposes.

### Synthesis of KIrO_2_ Nanoplatelets

The methodology of forming the potassium iridate nanoplatelets uses the intermediate potassium iridate formed via the solid-state reaction, as mentioned previously. The final powder formed is then spread evenly into an alumina crucible with a lid and annealed at 550 °C for 2 h under air conditions. The powder is left to cool overnight and then washed 3 times in DI water to remove the water-soluble byproducts and impurities. The final product is dried overnight at 75 °C.

### Synthesis of Hollandite-Type K_0.25_IrO_2_ Nanowires

Similar to the previous method, again, the formation of K_0.25_IrO_2_ nanowires uses the intermediate potassium iridate from the solid-state synthesis. The final powder formed is spread evenly into an alumina crucible (with a lid) and annealed at 750 °C for 2 h under air conditions. The powder is then left to cool and washed 3 times in DI water to remove the water-soluble byproducts and impurities. The sample is left to dry at 75 °C overnight for characterization purposes. The resulting powders are washed with DI H_2_O 3 times, to remove the water-soluble byproducts, and dried to form the solid product. The final yield of conversion from IrO_2_ to K_0.25_IrO_2_ was averaged to be 92.2%.

### Characterization Techniques

The visual morphology of the samples has been investigated using a Zeiss LEO Gemini 1525 field emission scanning electron microscope, operated at an accelerating voltage of 5 kV and working distances of <10 mm. Energy-dispersive X-ray spectroscopy (EDS) data were collected alongside the SEM images, using an Oxford x-act PentaFET Precision EDS detector, to enable compositional analysis. TEM imaging has been conducted using JEOL JEM-2100F and JEOL JEM-2100Plus electron microscopes operated at 200 kV accelerating voltage for the investigation of morphology and crystal structure, along with chemical analysis. Raman spectroscopy was conducted for chemical analysis, and a Raman Renishaw inVia Qontor confocal Raman microscope was used, at an excitation wavelength of 532 nm, using a 2400 lines mm^–1^ grating. XPS was conducted for further elemental and bonding analysis, using a high-throughput XPS machine, Thermo Fisher K-Alpha+. XRD patterns were obtained to investigate the crystallographic structure and composition of the samples, and an XRD Bruker D2 Phaser diffractometer was used (with the Cu source, the diffraction patterns were collected in the reflection scan geometry in the range 10–70° 2θ with a step size of 0.03° 2θ), and phase identification was conducted using “Match!”.

For all characterization, samples were drop-cast from the suspension (usually dispersed in DI H_2_O) onto a cleaned Si wafer and left to dry in an ambient environment until ready for examination, or the dry powder was measured directly.

### Electrochemical Tests

The electrochemical measurements were conducted using an RDE assembly (Pine Instruments Corporation) in a standard three-electrode glass cell (with a Luggin capillary), rotating between 16,000 and 2000 rpm. The electrolyte used throughout was 0.1 M HClO_4_, formed using 70 wt % HClO_4_ and diluted using ultrapure DI water. A catalyst-coated gold electrode (area: 0.1932 cm^2^) was used as the working electrode. Pt mesh was used as a counter electrode, and a hydrogen electrode (RHE) was used as a reference. All measurements were performed under ambient conditions at room temperature. *iR* correction was conducted via manually calculating the *iR* drop from the current and ohmic electrolyte solution resistance (*R*_u_).

Activity measurements were conducted via CV and LSV scans, with a voltage window of 1.4 V–1.7 V vs RHE, and scan rates at 20 mV/s. Electrochemical impedance spectroscopy was performed to calculate the Ru value, with a frequency range of 200 kHz to 10 mHz. Double-layer capacitance was calculated by multiple CV scans between either 0.85–0.95 V vs RHE or 0.75–0.85 V vs RHE, at scan rates of 500, 200, 100, 50, 20, 10, 5, and 1 mV/s. Stability tests were conducted following a method published by Murawski et al.,^55^ which includes extended CV cycling, with 15,000 cycles between 1.2 and 1.7 V vs RHE at a scan rate of 600 mV/s. Then, a reconditioning step was completed, with cycling CV scans at a lower potential of 0.6–1.4 V vs RHE, at a scan rate of 50 mV/s. Finally, the initial activity measurements were repeated, and percentage changes were calculated. The LSV scans were affected on average by an error of a few percentages.

Inductively coupled plasma–mass spectrometry (ICP–MS) was used to investigate catalyst degradation after cycling and quantifying losses. ICP–MS was calibrated prior to measurement, and four-point calibration curves were measured with a blank and the prepared standard solution of ^192^Ir. ^192^Ir was analyzed with a PerkinElmer NexION 2000c spectrometer, and data were recorded on Syngistix software. The recorded standard deviation of the ICP–MS measurements was from 0.001 to 0.01 ppb.

### Catalyst Ink Preparation and Deposition onto RDEs

To prepare the electrocatalyst ink, 1 mg of catalyst was added to 5 mL of 70% IPA/H_2_O, and this solution was sonicated for 10 min. 30 μL of 5 wt % Nafion was added, and the solution was further sonicated for 10 min. 10 μL of catalyst ink was drop-cast onto a cleaned Au insert and left to dry under a heat lamp for 1 h, giving a loading of 10.4 μm cm^–2^.

## Supplementary Material



## Notes

Experimental section; XRD and SEM images of K_*x*_Ir_*y*_Oz after different heating cycles; SEM images of intermediate K_*x*_Ir_*y*_O_*z*_ after heat treatments (550–750 °C); HR-TEM image of layered KIrO_2_ structures; XPS survey scans of KIrO_2_ platelets and K_0.25_IrO_2_ nanowires; LSV polarization curve of K_0.25_IrO_2_ nanowires compared to commercial IrO_2_; CV curves and double-layer capacitance values of KIrO_2_, K_0.25_IrO_2_, and commercial IrO_2_; iridium activity plots of IrO_2_, KIrO_2_, and K_0.25_IrO_2_ and Nyquist plots of the corresponding samples; XRD spectra of K_0.25_IrO_2_ and KIrO_2_ before and after electrochemical testing; ICP–MS plots of IrO_2_, KIrO_2_, and K_0.25_IrO_2_; XANES region of KIrO_2_ and K_0.25_IrO_2_ before and after electrochemical tests (PDF)
