# Supplementary material for: Crystal-Phase Engineering of Nanowires and Platelets of K x IrO2 for Efficient Water Oxidation
Source: ACS Mater Au. 2025 Oct 15;5(6):1070–9. doi: 10.1021/acsmaterialsau.5c00127 (PMC12616440; doi:10.1021/acsmaterialsau.5c00127)
Supplement: Supplementary file 1 [file mg5c00127_si_001.pdf]

Supplementary Information

Crystal-phase engineering of nanowires and platelets of K_xIrO_2 for efficient water oxidation

Rachael Quintin-Baxendale[a], Maria Sokolikova[a], Yemin Tao[a], Evan Fisher[a], Nagaraju Goli[a], Haoyu Bai[a], James Murawski[a], Guangmeimei Yang[a], Veronica Celorrio[b,c,d], Caiwu Liang[a,e], Reshma R. Rao[a,e], Ifan E.L. Stephens[a]*, Cecilia Mattevi[a] *.

[a] *Department of Materials, Imperial College London, London, SW7 2AZ, UK*

[b] *Diamond Light Source Ltd., Harwell Science and Innovation Campus, Chilton, Didcot OX11 0DE, United Kingdom*

[c] *Department of Chemistry, University College London, 20 Gordon Street, London WC1H 0AJ, United Kingdom*

[d] *UK Catalysis Hub, Research Complex at Harwell, Rutherford Appleton Laboratory, Harwell Oxon, Didcot OX11 0FA, United Kingdom*

[e] *Grantham Institute‒Centre for Climate Change and the Environment, Imperial College London, South Kensington Campus, London SW7 2AZ, U.K.*

Corresponding authors

*i.stephens@imperial.ac.uk

*c.mattevi@imperial.ac.uk

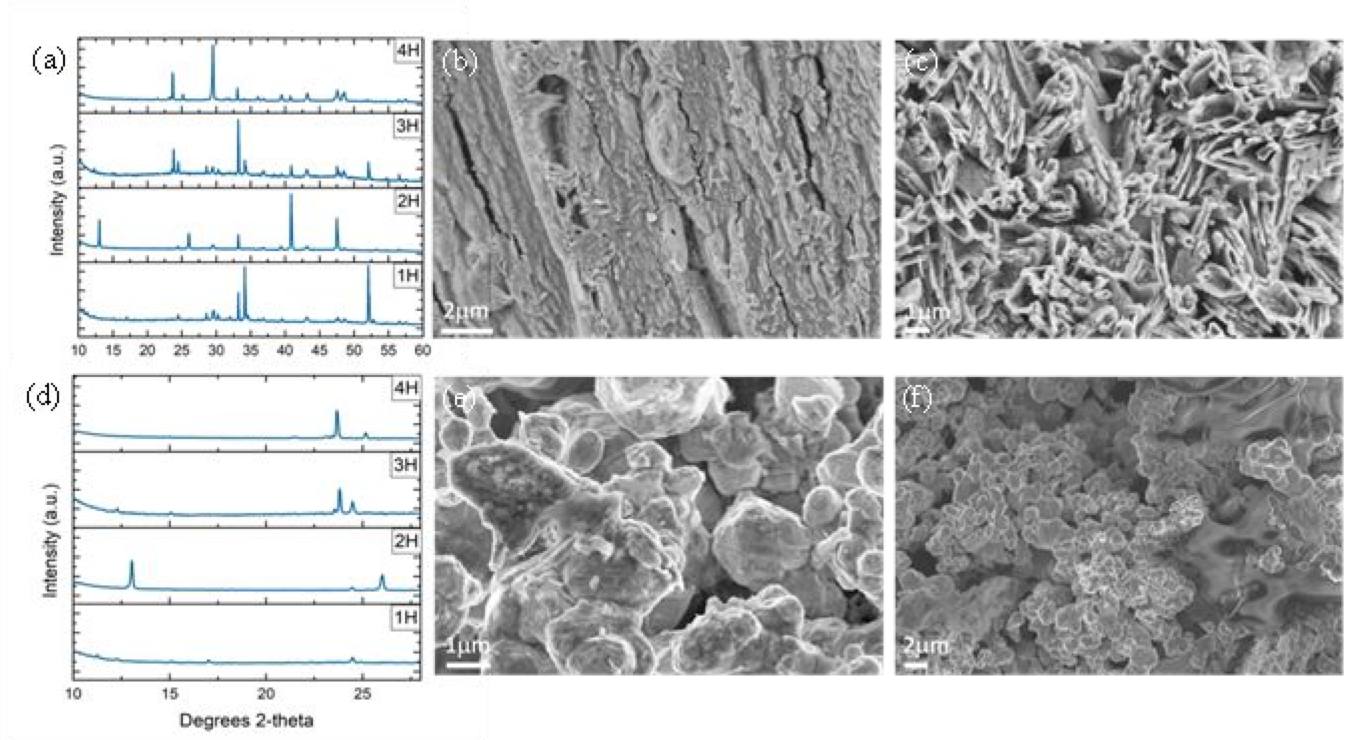

Figure S1. *Intermediate K_xIr_yOz formed upon different heating cycles. (a) XRD pattern from 10°-60° showing heat cycles 1H-4H, with (d) XRD pattern from 10°-30° showing heat cycles 1H-4H. In both (a) and (d), the only synthesis showing both peaks at 13° and 26° is the 2H heat cycle, highlighting the (001) and (002) peaks respectively. Figures S1 (b), (c), (e) and (f) show SEM images of the compounds formed after 1H, 2H, 3H and 4H respectively, and a layered structure emerges at heat cycle 2H.*

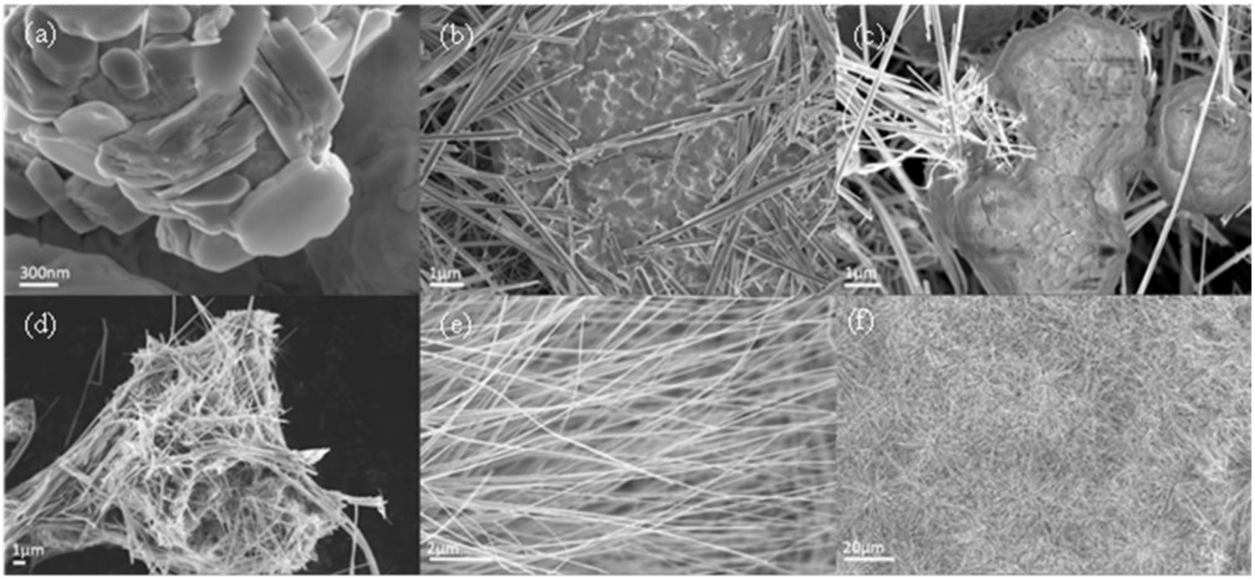

Figure S2. *SEM images of intermediate $K_xIr_yO_z$ after different heat treatments (2 hours) under air conditions. (a) 550°C; (b) 600°C; (c) 650°C; (d) 700°C; (e) and (f) 750°C. Nanoplatelets are clearly seen at 550°C, and uniform nanowires are seen at 750°C. Temperatures in-between show a slow progression from complete nanoplatelets to complete nanowires.*

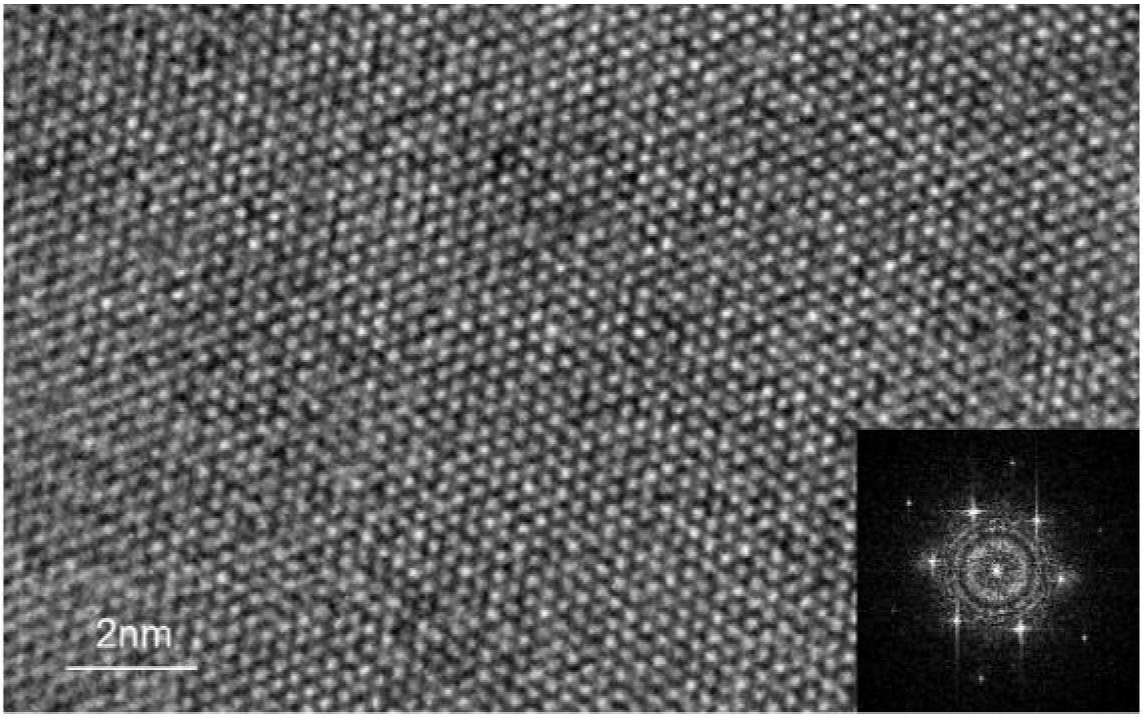

Figure S3. *High resolution TEM image of layered $KIrO_2$ structure. Inset: accompanying diffraction pattern.*

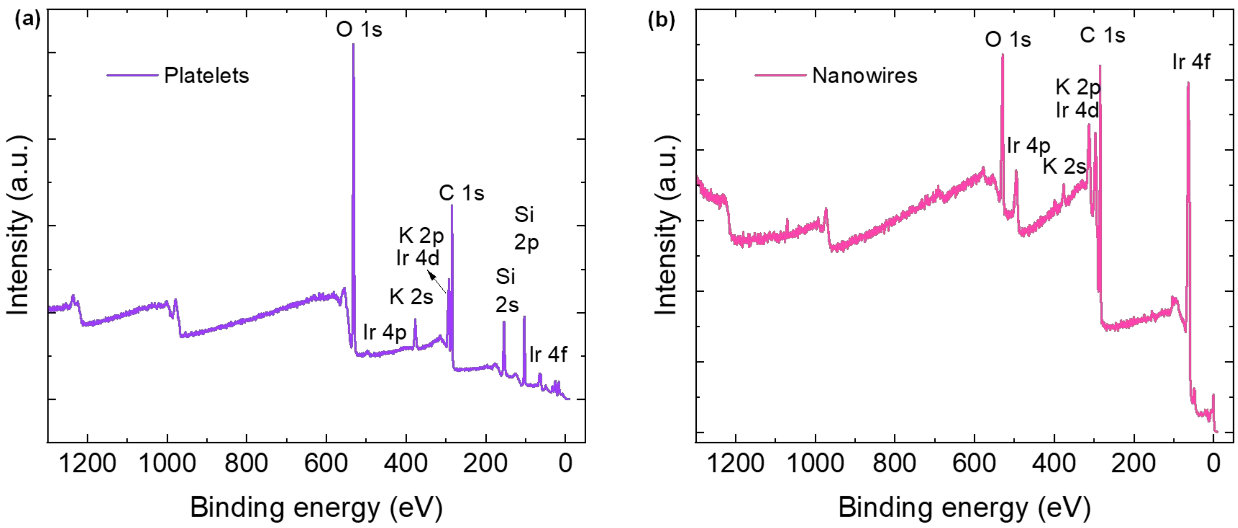

Figure S4. *XPS survey scan spectra of (a) $KIrO_2$ platelets and (b) $K_{0.25}IrO_2$ nanowires. In the main manuscript of Figure 2, the area ratio of Ir^{IV} to Ir^{III} peaks is ~3:1, corresponding to an overall iridium oxidation state of ~3.75. In the peak fitting of Figure 3 XPS data, the peak fitting was performed using only Ir^{III}. Peak fit parameters are based on prior reports on iridium oxides and analogous structures[3,4].*

In Figure S5a, the effect of number of washes on the current density is shown. The first 2 washes can be seen to significantly increase the current density, owing to the reduction in inactive precursors that remain in the final product. A slight increase is seen in the third wash, corresponding to the final removal of the product. Washes are kept to a minimum to avoid potential fracture and agglomeration of nanowires. This effect is shown in Figure S5c.

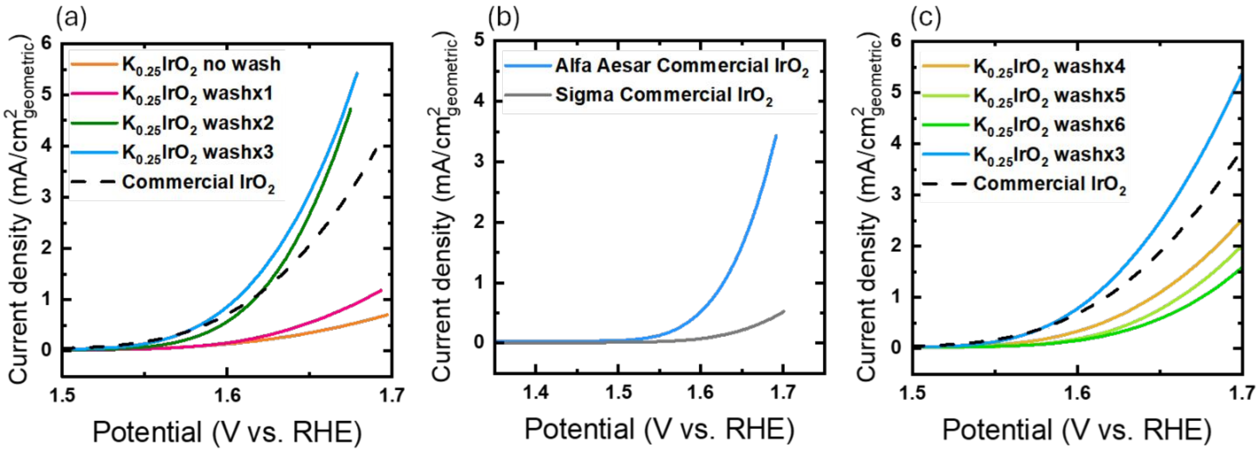

Figure S5. *(a) LSV polarisation curve (scan rate: 10mV/sec) showing the current density achieved from $K_{0.25}IrO_2$ nanowires after various numbers of washes of final stage of synthesis, with dramatic activity increase by 3 wash numbers. (b) LSV polarisation curve (scan rate: 10mV/sec) comparing two types of commercial IrO_2. One was a Sigma Commercial IrO_2 while the second was a commercial optimised IrO_2 by Alfa Aesar which was used as the commercial sample throughout; these show the dramatic difference in activity between two rutile IrO_2 samples. (c) Showing the effect of an increased number of washes on the performance of the samples in comparison to the Alfa Aesar commercial IrO_2 at a scan rate of 10 mV/s. The 3 wash sample from S5 (a) is shown as a comparison.*

Figure S5 show polarisation curves, from 1.3-1.7V vs. RHE, for unwashed, 1, 2 and 3 washes (via centrifugation in DI water) This data clearly shows the dramatic increase in activity seen after 2 and 3 washes, which will be due to the washing and subsequent removal of inactive by-products formed during the synthesis. However, less expectedly, Figure S5c shows that the activity again

declines at a higher number of washes, proving that 3 washes is the optimal option. Hypothesising why this could be the case, as it is understood the high stability of the $K_{0.25}IrO_2$ compound, it is unlikely that the washing is affecting the stoichiometry of the material. Therefore, the reducing activity could be due to the force of the washing cycles causing a breakdown in the length of the wires, along with promotion of clustering and agglomeration, in turn reducing electrochemical surface area.

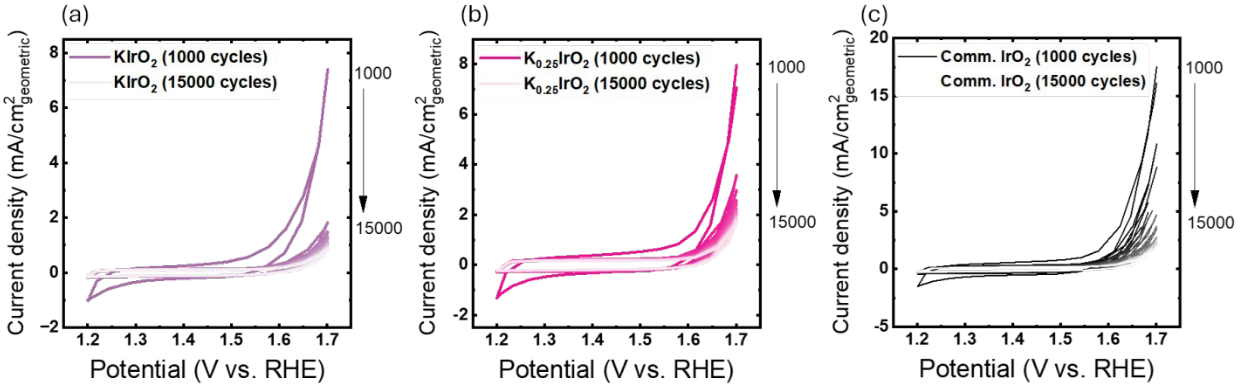

Figure S6. *Part of AST: Extended CV scans from 1.2-1.7 V vs RHE at a scan rate of 600mV/sec for 15,000 cycles. (a) $KIrO_2$; (b) $K_{0.25}IrO_2$; (c) AA IrO_2.*

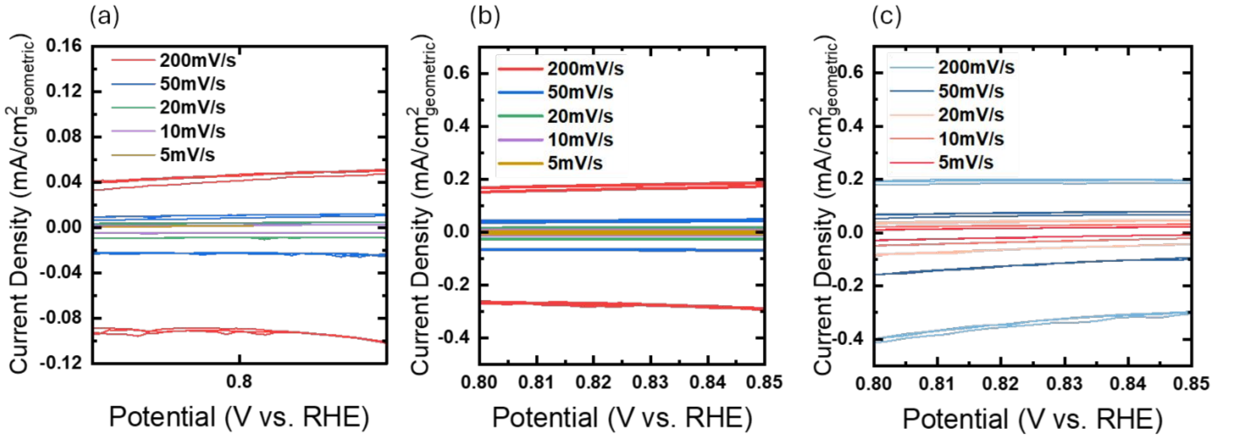

Figure S7. *CV scans run at various scan rates between set potentials, used to determine double layer capacitance values. (a) commercial* IrO_2 *(*C_{dl}*: 2.53μF); (b)* $K_{0.25}IrO_2$ *(*C_{dl}*: 2.25μF); (c)* $KIrO_2$ C_{dl}*: 0.67μF).*

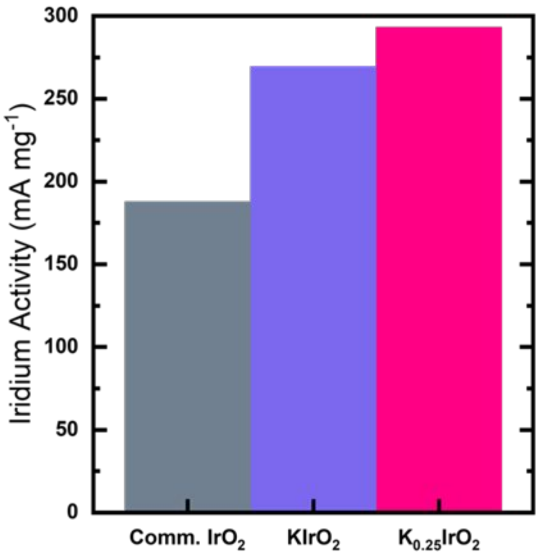

Figure S8. *The activity of commercial* IrO_2*,* $KIrO_2$ *and* $K_{0.25}IrO_2$ *normalised to the amount of iridium contained within the sample. By potassiating the structure, the overall iridium loading is reduced, giving further enhanced activity.*

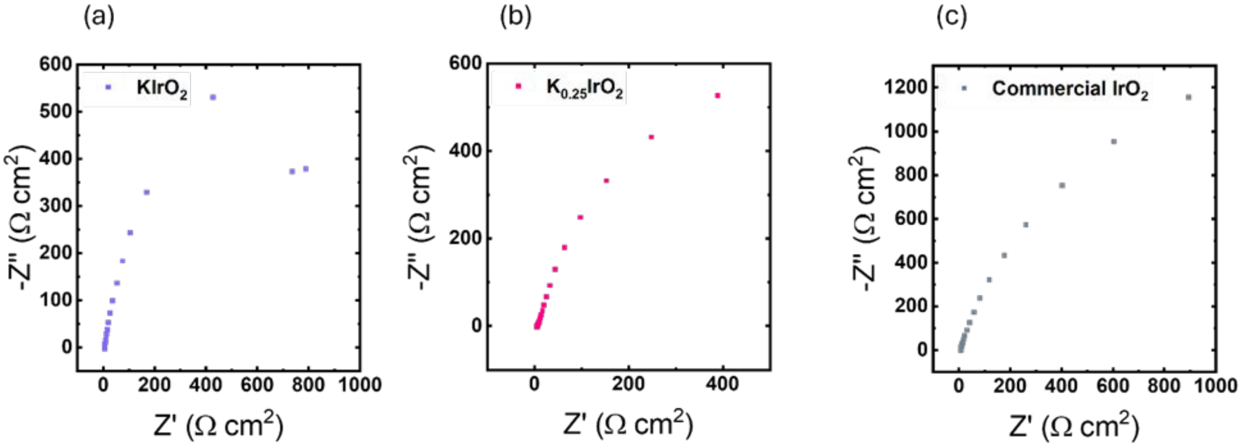

Figure S9. *Nyquist plots created from EIS measurements, used to calculate Rs values, for iR compensation. (a)* $KIrO_2$*; (b)* $K_{0.25}IrO_2$*; (c) commercial* IrO_2*.*

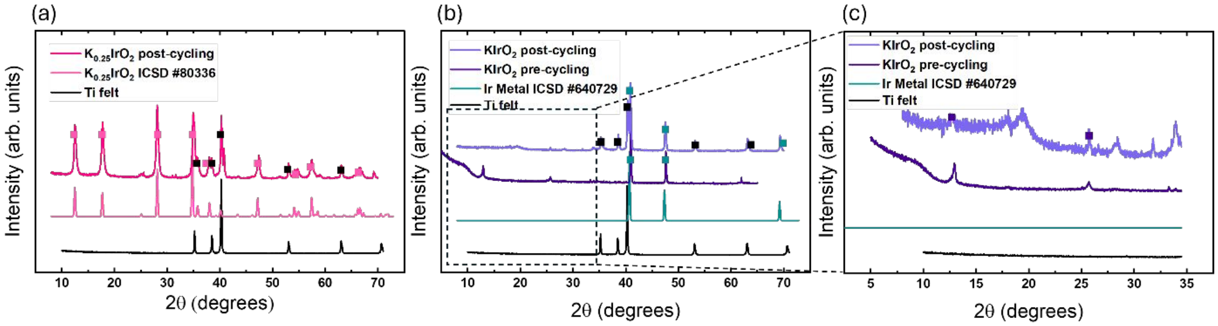

Figure S10. *XRD of (a) $K_{0.25}IrO_2$ after 50 cycles from 1.2 to 1.7 V compared to the Inorganic Crystal Structure Database (ICSD) for the hollandite iridate. $K_{0.25}IrO_2$ was measured from 8° to 70° 2θ and Ti felt was measured from 10° to 75° 2θ on a Bruker D2 Phaser. XRD of (b) $KIrO_2$ before and after 50 cycles from 1.2 to 1.7 V. XRD of $KIrO_2$ post-cycling was taken from 8° to 70° 2θ on a Bruker D2 Phaser, Ti felt from 10° to 75° on a Bruker D2 Phaser, and $KIrO_2$ pre-cycling between 5° and 65° 2θ on an MPD Panalytical as the raw sample powder. (c) shows a snapshot of the low-angle region of $KIrO_2$ diffraction with $KIrO_2$ samples before and after cycling. All ICSD patterns are extended from 0° to 75° 2θ.*

The XRD patterns were taken to investigate the crystallographic structure and composition of the samples, and an XRD Bruker D2 Phaser diffractometer was used (with the Cu source, the diffraction patterns were collected in the reflection scan geometry in the range 8-70° 2θ with a step size 0.03 °2θ), and phase identification was conducted using 'Match!'. Patterns were collected pre- and post- electrochemical activity testing (50 CV cycles from 1.2-1.7V vs. RHE), to understand any changes to crystal structure. The post cycling samples were tested on titanium foil,

resulting in a significant response in the diffraction pattern. In the $K_{0.25}IrO_2$ sample, both pre- and post- electrochemical testing, there is a clear match to the ICSD pattern, leading to a high confidence in the maintenance of the structure of the nanowires. There is a minor shift in some peaks, however due to the nature of the nanowires, this could be due to peak broadening, and the calculated lattice parameters see only a 0.1 Å a-axis reduction, and a 0.5° increase in the β angle from the database pattern, a minor shift that could arise from errors in the mounting of the sample.

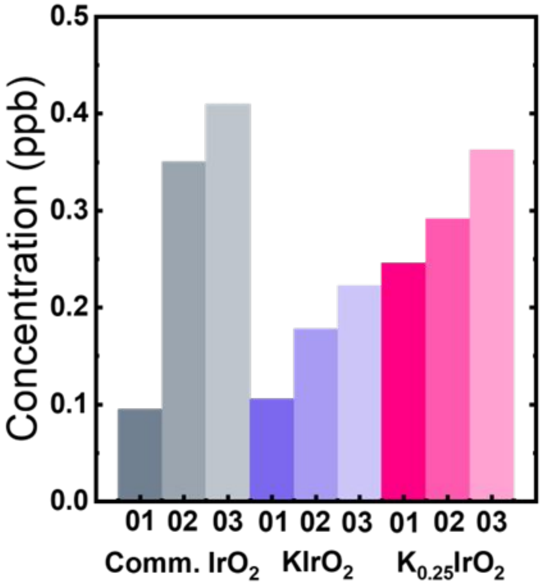

Figure S11. *ICP MS data for pre (01) during (02) and post (03) accelerated stress test (AST).*

For $KIrO_2$, the substrate of titanium felt dominates the pattern, along with peaks that are attributed to iridium metal. At low angles, were the Ti and Ir response is minimal, there are small peaks that seem to be maintained through cycling, however several new peaks raise the possibility of alternative species, such as IrOOH, being formed. In this case, the peaks from structural changes will change. Further analysis is required to understand if the low angle peaks seen post-cycling originate from KIrO2, and what they represent.

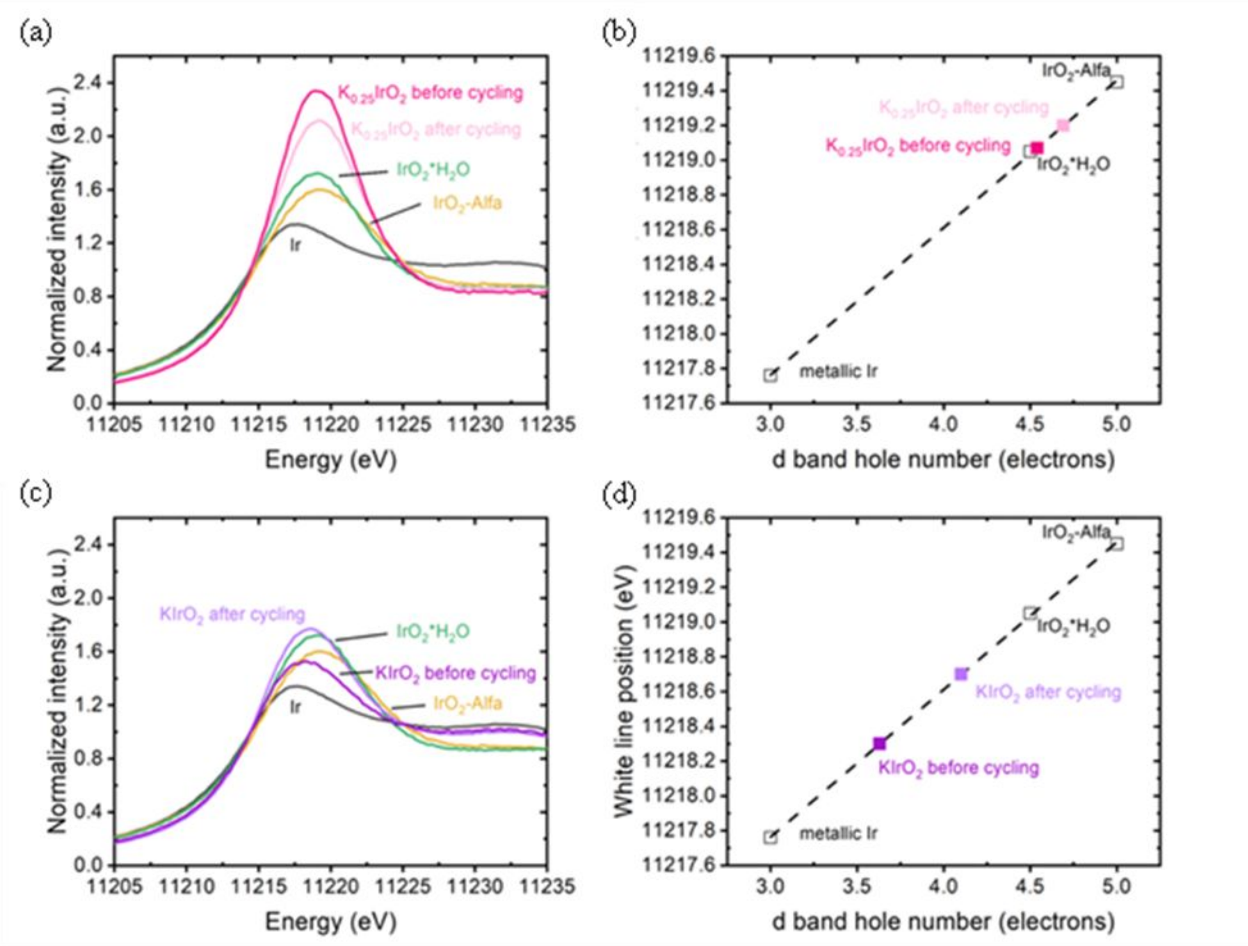

Figure S12. *Ir L3-edge XANES region of the reference standard samples and (a) $KIrO_2$ (c) $K_{0.25}IrO_2$ before and after electrochemical tests; (b)(d) are the white line position as a function of the formal d-band hole number. The corresponding formal d-band hole number for metallic iridium, IrO_2*H_2O and IrO_2 were 3, 4.5 and 5. The data reported above were all measured in the same period at B18 Diamond Light Source, UK.*

Before cycling, $K_{0.25}IrO_2$ shows an average d-band hole number of around 4.5, corresponding to an average oxidation state of around 3.5. After cycling, Ir oxidation state slightly changes, increasing to around +3.7 (4.7 d-band hole). Before cycling, $KIrO_2$ showed an average d-band hole number of around 3.6, corresponding to an average oxidation state lower than +3. After cycling, Ir oxidation state is around +3 (4.1 d-band hole). To note, the uncertainty of the typical XAS measurements and corresponding data analysis is around 0.1-0.2 eV.

Table S1. Parameters used in XAS analysis software Athena for EXAFS analysis.

	Rbkg value	Forward Fourier Transform Parameters: k range	Backward Fourier Transform Parameters: R range
Pristine $KIrO_2$	1	3 to 10	1 to 6
$KIrO_2$ after cycling	1	3 to 10	1 to 6
Pristine $K_{0.25}IrO_2$	1	3 to 10	1 to 6
$K_{0.25}IrO_2$ after cycling	1	3 to 10	1 to 6

All the EXAFS data analysis is processed using Athena Software, using the same parameters for the Fourier Transform. The fitting of the EXAFS data was particularly challenging due to the numerous possible scattering paths involved, which is much more complex compared to typical rutile iridium oxides. We thus report the raw data after Fourier Transform in the main text without fitting.

SI References

[1] Takimoto, D.; Fukuda, K.; Miyasaka, S.; Ishida, T.; Ayato, Y.; Mochizuki, D.; Shimizu, W.; Sugimoto, W. Synthesis and Oxygen Electrocatalysis of Iridium Oxide Nanosheets. *Electrocatalysis* **2017**, *8* (2), 144–150. https://doi.org/10.1007/s12678-016-0348-4.

[2] Murawski, J.; Scott, S. B.; Rao, R.; Rigg, K.; Zalitis, C.; Stevens, J.; Sharman, J.; Hinds, G.; Stephens, I. E. L. Benchmarking Stability of Iridium Oxide in Acidic Media under Oxygen Evolution Conditions: A Review: Part I: Probing Degradation of Iridium-Based Oxide Catalysts. *Johnson Matthey Technology Review* **2024**, *68* (1), 121–146. https://doi.org/10.1595/205651323X16848455435118.

[3] Sun, W.; Song, Y.; Gong, X.-Q.; Cao, L.; Yang, J. Hollandite Structure $K_{x\approx 0.25}IrO_2$ Catalyst with Highly Efficient Oxygen Evolution Reaction. *ACS Appl. Mater. Interfaces* **2016**, *8* (1), 820–826. https://doi.org/10.1021/acsami.5b10159.

[4] Pfeifer, V.; Jones, T. E.; Velasco Vélez, J. J.; Massué, C.; Arrigo, R.; Teschner, D.; Girgsdies, F.; Scherzer, M.; Greiner, M. T.; Allan, J.; Hashagen, M.; Weinberg, G.; Piccinin, S.; Hävecker, M.; Knop-Gericke, A.; Schlögl, R. The Electronic Structure of Iridium and Its Oxides. *Surface & Interface Analysis* **2016**, *48* (5), 261–273. https://doi.org/10.1002/sia.5895.
